# Hazardous Alcohol Consumption Among High-School Teachers in Bahir Dar, Ethiopia

**DOI:** 10.1016/j.focus.2025.100347

**Published:** 2025-04-14

**Authors:** Abrham Maru Moges, Gizachew Asnake Tiruneh, Tilahun Belete Mossie

**Affiliations:** 1 Department of Psychiatry, Teda Health Science College, Gondar, Ethiopia; 2 Department of Epidemiology, Teda Health Science College, Gondar, Ethiopia; 3 Department of Psychiatry, College of Medicine and Health Sciences, Bahir Dar University, Bahir Dar, Ethiopia

**Keywords:** Hazardous alcohol consumption, alcohol consumption, high-school teachers, Ethiopia

## Abstract

**Introduction:**

Screening teachers for hazardous alcohol consumption is important to designing interventions that promote a conducive teaching–learning environment. However, there is scarce evidence on screening activities among teachers in low-income countries, particularly in Ethiopia. This study aimed to assess the prevalence of hazardous alcohol consumption and associated factors among high-school teachers in Bahir Dar city, Ethiopia.

**Methods:**

An institution-based cross-sectional study was conducted in randomly selected 407 teachers. Hazardous alcohol consumption was assessed using the Fast Alcohol Screening Test, with a score ≥3 indicating a positive result. Data were collected electronically using EpiCollect5 through interviewer administration. SPSS (Version 25) was used for data analysis. Bivariable and multivariable logistic regressions were carried out, and variables with a *p*<0.05 in the final analysis were considered significantly significant.

**Results:**

The prevalence of hazardous alcohol consumption was 16% (95% CI=12.40, 19.54): 20.9% among men and 6.1% among women. The odds of hazardous drinking were higher among male participants (AOR=4.46, 95% CI=1.87, 10.60), among teachers aged <41 years (AOR=3.76, 95% CI=1.39, 10.1.5), among first-degree holders than among second-degree holders (AOR=2.72, 95% CI=1.14, 6.49), and among those who screened positive for psychological distress (AOR=4.28, 95% CI=2.15, 8.50). In addition, participant with low social support had 3.95 times higher odds of hazardous alcohol consumption (AOR=3.95, 95% CI=1.49, 10.45).

**Conclusions:**

Hazardous alcohol consumption was high in teachers, particularly when compared with previous literature examining hazardous alcohol consumption in the general population. This refers to integrating lifestyle and other psychosocial interventions in the teachers’ development programs to prevent alcohol consumption, with more emphasis on younger employees, men, those who experience psychological distress, and those who lack close interpersonal relationships.

## HIGHLIGHTS

• A 16% prevalence of hazardous alcohol consumption was found among high school teachers in Bahir Dar.
• Hazardous alcohol consumption was more prevalent among male, younger, and lower educational status teachers.
• Psychological distress was associated with 4.28 times higher odds of hazardous alcohol consumption.
• The risk of hazardous alcohol consumption was heightened by low social support.
• There is a need to integrate lifestyle and psychosocial interventions into teachers' programs.

## INTRODUCTION

Alcohol consumption is a public health issue that is widely practiced both in the general public and at the workforce. It is also one of the major risk factors for health-related problems worldwide.^1^ According to the 2022 National Survey on Drug Use and Health, 28.8 million adults experienced alcohol use disorders in 2021.^2^

Hazardous alcohol consumption (HAC) is a quantity or pattern of alcohol consumption that puts the user or others at risk of undesirable consequences.^3^ Persistent heavy alcohol consumption causes physical, psychological, social, and financial problems. A standard drink is defined as 10 grams of alcohol, and consumption becomes problematic when this amount is exceeded. HAC can be identified through various indicators, such as level of alcohol use (>6 drinks on an occasion for women, >8 drinks on an occasion for men), gaps in memory caused by alcohol use, failure to maintain normal responsibility due to alcohol use, or whether someone else (a relative, a friend, a teacher, or others) expresses concern about a person’s alcohol use.^3^

HAC can be related to different factors. For instance, the level of education attained plays an important role in protecting individuals from engaging in HAC.^4^ According to numerous prevalence studies, excessive alcohol consumption is common among teachers.^5^^,^^6^ Some studies have shown that teachers drink more alcohol than the general population.^7^ Approximately 25% of head teachers in Britain were reported to engage in heavy alcohol consumption. In Texas, a survey of 500 teachers revealed that approximately 50% of teachers frequently used alcohol.^8^ A study performed in a general outpatient population in South Africa indicated that 41.2% of men and 18.3% of women were identified for HAC.^9^ Regarding alcohol use in Ethiopia, certain demographic characteristics are known to be risk factors, such as living in rural areas or being male; however, less is known about the drinking behavior of Ethiopian teachers. Prevalence studies performed in different parts of Ethiopia have shown that HAC was 3.7% and 2.7% in rural and urban areas, respectively.^10^ A pooled prevalence study in Ethiopia revealed that the prevalence of HAC was 8.94% and was higher in men (11.58%) than in women (1.21%).^1^

The teacher’s responsibility is to educate and mentor students and outline their values, in addition to providing knowledge. Their health and well-being are impacted by their behaviors, which may impact their duties as role models for youngsters.^11^ They are the frontline of child and adolescent alcohol prevention activities, and as the family structure and function continue to change, schools are taking on more responsibility for socialization. Teachers who engage in HAC may find it more difficult to fulfill these obligations. The message about HAC prevention being taught in the classroom can be questioned if teachers have problems with HAC.^12^ Because they function as role models for their pupils and the general public, their drinking habits should receive special attention.^13^ Their role in the prevention of alcohol consumption involves modeling alcohol-free or limited alcohol-intake behavior.^14^

Tella (local beer), Tej (local honey-wine), and Areki (local liquor) are the common and popular traditional (homemade) alcoholic beverages in Ethiopia. There is no clear local alcohol content policy for each alcoholic beverage in Ethiopia. Previous local studies revealed that Tella has an average alcohol value of 4% (2%–6%), that of Tej is 11% (8.9%–13.2%), and that of Areki is 37% (33.95%–40%).^15^, ^16^, ^17^ Other factory products of beer, wine, and liquor are also consumed mainly in urban areas.

Prior findings revealed that teachers engaged in HAC owing to different reasons, including peer pressure and environmental, personal, and social factors. Peer pressure and environmental factors are the leading causes of alcohol drinking^,^.^18^ The presence of known chronic medical illness such as cancer, cardiovascular disease, or diabetes and family history of mental illness were also associated to HAC.^19^, ^20^, ^21^ Culture, religion, and work can also influence drinking behavior.^22^

In addition to direct harm, such as liver disease, HAC is a major risk factor for injury, violence, and other risky behavior. All these, in general, lead to poor performance, deterioration in one’s well-being, morbidity to different chronic illnesses, and even death.^23^

Study hypothesis was that there would be a significant association between HAC in teachers in Ethiopia and 1 or more of the sociodemographic, medical, psychosocial, and behavioral factors assessed. This study sought to determine the prevalence of HAC among high-school teachers in Bahir Dar City, Northwest Ethiopia.

## METHODS

### Study Sample

An institutional based cross-sectional study was conducted in Bahir Dar City, the capital of the Amhara Region, Ethiopia. The city is located 565 km Northwest of Addis Ababa, the capitals of Ethiopia. According to data received from the city mayor's office, the education system is divided into primary (1st–8th grade) and high-school (9th–12th grade) education. The Bahir Dar municipal education office reported that there were 2,048 public (government) teachers in Grades 1–12 in the city, of whom 847 were high school (9th–12th grade) teachers working in 11 high schools (unpublished data from Bahir Dar municipality). The study was conducted from June to July 2022.

All public high-school teachers at Bahir Dar City who were available at their workplace at the time of data collection were included in the study.

Teachers who were unable to provide accurate information during data collection were excluded.

The sample size was determined using a single population proportion formula with the assumption of 95% CI, 5% margin of error, and 50% proportion. The calculated sample size for the study was 384; thus, by adding 10% for the possible nonresponse rate, the final sample size included 423 participants.

**Figure 1:**
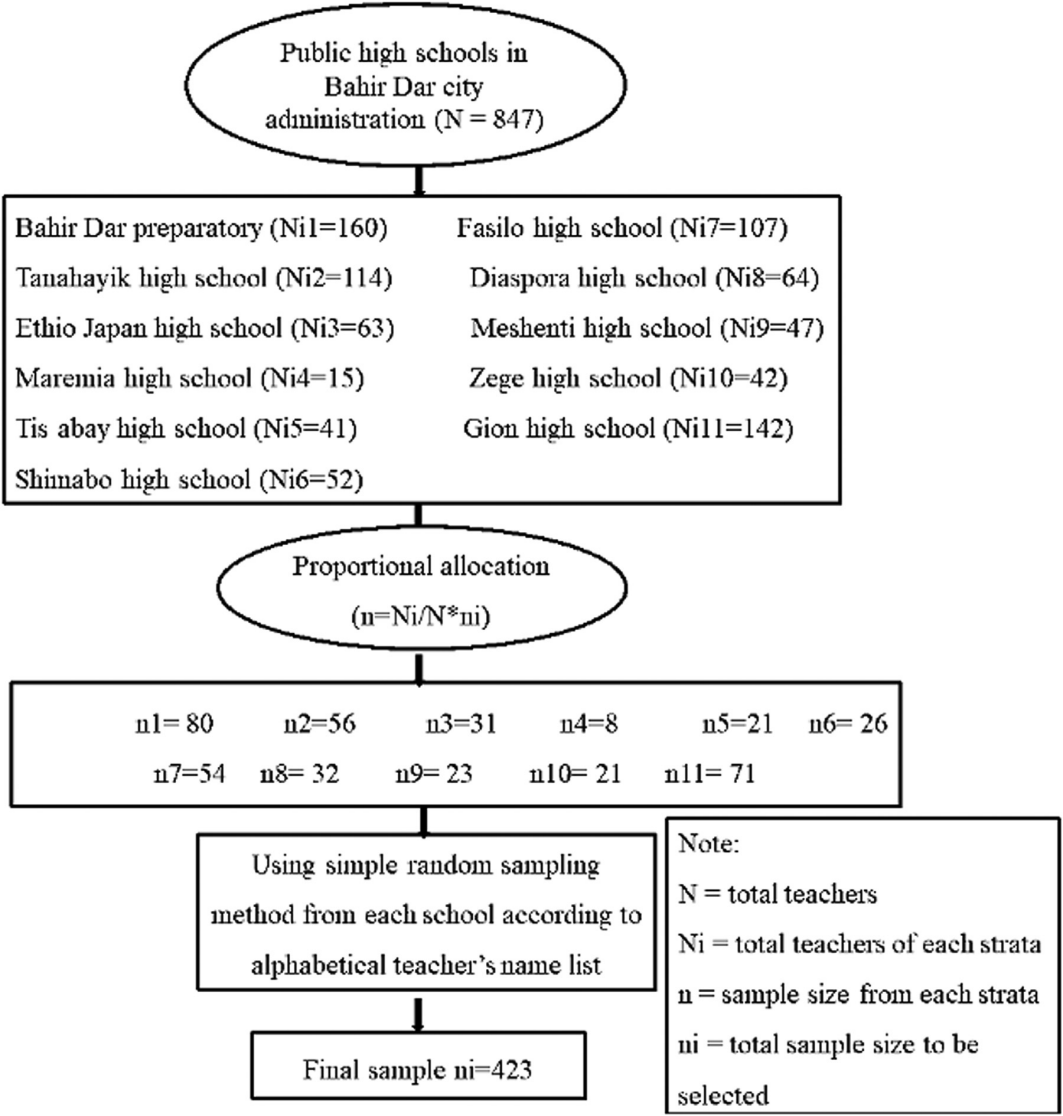
Schematic representation of the sampling procedure.

There were 11 public high schools and 847 high-school teachers in the Bahir Dar City. Study participants were recruited from all the high schools. The complete name lists of teachers were collected from the public high schools, and all the teachers were alphabetically ordered. The authors selected study participants by a simple random sampling method from each high school according to their proportion (Figure 1).

### Measures

HAC was the outcome variable. Sociodemographic variables (age, sex, marital status, number of children, religion, educational states, years of experience, living arrangement), psychosocial variables (psychological distress, social support, teachers who have friends who consume alcohol, teachers who take alcohol with their friends, family history, presence of known chronic medical conditions, stressful life events), and behavioral (physical exercise, current cigarette smoking, current khat user) characteristics were independent variables.

Data were collected through interviewer-administered electronic data collection using EpiCollect5. The data collection tools consisted of sections covering HAC, sociodemographic characteristics, psychosocial factors, and behavioral factors. Four diploma nurses were recruited as data collectors, and 1 psychiatry nurse served as facilitator. The investigators supervised the data-collection process, ensuring adherence to ethical guidelines. Participants were informed about the voluntary natures of their participation, the purpose of the study, and its importance of providing accurate information.

The Fast Alcohol Screening Test (FAST) was used to screen for HAC. FAST is a brief screening questionnaire for HAC, consisting of 4 questions that can be administered in a minute or less. It was derived from the Alcohol Use Disorders Identification Test. A score of 3 or more of 16 indicates HAC.^24^ The FAST has demonstrated strong psychometric properties, with a sensitivity of 0.93 and specificity of 0.88.^25^ Concurrent validity of Alcohol Use Disorders Identification Test tool was achieved in 2022 in Ethiopia,^26^ and FAST has been used in a prior study in Ethiopia.^27^ When local alcoholic beverages were reported by the respondents using various receptacles, they were converted into standard equivalent alcohol units. Estimates of the alcohol content of the various locally accessible beverages were previously determined according to the WHO standard drink (10 grams), which was subsequently used to calculate the number of alcohol units consumed.^26^

The Kessler Psychological Distress Scale (K10) was used to assess psychological distress.^28^ The scale consists of 10 questions assessing distress experience over the past 4 weeks. Responses were scored on a 5-point ordinal scale, reflecting how often participant experienced symptoms over the past 4 weeks. Each item on the scale is rated from 1 to 5, ranging in severity from none of the time to all of the time. The total K10 score for each participant was obtained by summing all 10 items, yielding a range from 10 to 50. In line with prior research, participants with a score ≥22 were considered positive for psychological distress.^29^ The K10 has demonstrated reliability and validity in the previous research, with a Cronbach’s alpha coefficient of 0.91.^30^ The K10 scale has also been validated in Ethiopia,^31^ demonstrating strong internal consistency (0.93), a sensitivity of 84.2%, and a specificity of 77.8%.

The Oslo Social Support Scale was used to assess the level of social support teachers received from others. The scale categorizes the level of social support into 3 levels: poor social support (3–8), moderate social support (9–11), and high social support (12–14).^32^

The List of Threatening Experiences was used to assess stressful life experiences over the past 6 months. The scale contains 12 questions about stressful life events dichotomous with the no or yes response options, where a single yes may indicate HAC.^33^ List of Threatening Experiences has been used in a prior study in Ethiopia.^34^^,^^35^

### Statistical Analysis

The collected data were exported to SPSS (Version 25.0) for analysis. The results are presented using descriptive statistics. To identify the factors associated with HAC, the authors performed bivariable and multivariable analyses. The bivariable analysis was carried out to select variables for the multivariable analysis. Variables with a *p*<0.25 were selected for the final analysis. In the multivariable analysis, statistical significance was determined at *p*<0.05.

## RESULTS

A total of 407 of the 423 teachers provided complete responses, yielding a response rate of 96%. Two hundred and sixty-five participants were male (64.9%), with the youngest participant being aged 24 years. Of the participants, 81.3% were married, and 68.8% held a bachelor’s degree (Table 1). The overall prevalence of HAC (using a FAST score ≥3) was 16% (95% CI=12.40, 19.54%), with higher rates among male (20.9%) than among females (6.1%). The psychosocial and behavioral characteristics of the participants are summarized in Table 2.

**Table 1:** Sociodemographic Characteristics of Participants, Bahir Dar City Public High-School Teachers, Northwest Ethiopia, 2022

Characteristics	*n*
Total	N=407
Sex	
Male	264 (64.9%)
Female	143 (35.1%)
Age group, years	
<40	272 (66.8%)
41–60	135 (33.2%)
Religion	
Orthodox	374 (91.9%)
Muslim	23 (5.6%)
Protestant	10 (2.5%)
Marital status	
Married	331 (81.3%)
Single	76 (18.7%)
Number of children	
No children	91 (22.4%)
1–3 children	264 (64.8%)
≥4 children	52 (12.8%)
Education status	
Degree	280 (68.8%)
Masters	127 (31.2%)
Living arrangement	
Living with family	317 (77.9%)
Living with alone	85 (20.9%)
Living with friends	5 (1.2%)
Years of experience	
1–10	33 (8.1%)
11–20	311 (76.4%)
≥21	63 (15.5%)

*Note: n* denotes the number of participants (%).

**Table 2:** Psychosocial and Behavioral Characteristics of Participants, Bahir Dar City High-School Teachers, Northwest Ethiopia, 2022

Variables	*n*
Total participants	N=407
Experience psychological distress	85 (20.9%)
One or more experience of stressful life events	145 (35.6%)
Sedentary or weak in physical exercise	384 (94.3%)
Current khat user	14 (3.4%)
Current cigarettes smoker	15 (3.7%)
Teachers who have friends who take alcohol	125 (30.7%)
Teachers who take alcohol with their friends	82 (20.1%)
Family history of mental illness	11 (2.7%)
Social support	
Poor social support	105 (25.8%)
High social support	121 (30%)
Known chronic medical conditions	
Diabetes	6 (1.5%)
Hypertension	1 (0.2%)
Kidney Disease	3 (0.7%)
Heart disease	2 (0.5%)

*Note: n* denotes the number of participants (%).

The result of logistic regression analysis of the different factors and their association with HAC are presented. In the bivariable logistic regression analysis, sex, age, educational status, number of children, work experience, currently khat user, current cigarette smoker, social support, psychological distress, physical exercise, stressful life events, teachers who have friends who take alcohol, and teachers who consume alcohol with their friends were associated with HAC (*p*<0.25). However, in the multivariable logistic regression, sex, age, education level, social support, and psychological distress were significantly associated with HAC among public high-school teachers working in Bahir Dar City (*p*<0.05). The Hosmer–Lemeshow test for model fit was not significant for this multivariable model (*p*=0.375), indicating that the data fit the model well (Table 3).

**Table 3:** Multivariable Logistic Regression

	Hazardous alcohol consumption		OR	
Characteristics	Yes	No	Unadjusted OR (95% CI)	Adjusted OR (95% CI)
Sex				
Male	57	218	4.05 (1.87, 8.77)	**4.46 (1.87, 10.60)**
Female	8	124	ref	ref
Educational status				
Degree	55	225	2.86 (1.41, 5.82)	**2.72 (1.14, 6.49)**
Masters	10	117	ref	ref
Social support				
Low social support	30	91	3.99 (1.74, 9.17)	**3.95 (1.49, 10.45)**
Moderate social support	27	154	2.12 (0.92, 4.87)	2.39 (0.92, 6.15)
High social support	8	97	ref	ref
Psychological distress				
Psychological distress	31	54	4.86 (2.76, 8.57)	**4.28 (2.15, 8.50)**
No psychological distress	34	288	ref	ref
Age, years				
20–40	57	215	4.21 (1.95, 9.11)	**3.76 (1.39, 10.15)**
41–65	8	127	ref	ref

*Note*: Boldface indicates statistical significance (*p*<0.05).

## DISCUSSION

This study primarily determined the prevalence of HAC and the factors associated with it among high-school teachers in Bahir Dar City, Ethiopia. The finding will be helpful to different actors engaged in education, alcohol prevention, and teachers’ well-being, which require significant attention of policy makers and stakeholders. Sex, age, educational status, social support, and experiencing psychological distress were associated with HAC among public high-school teachers.

The prevalence of HAC in this study was 16% (95% CI=12.40, 19.54%). This is consistence with the findings of studies conducted among teachers in Japan (14.9%)^36^ and hospital doctors in Germany (19.8%).^37^ However, it is lower than studies conducted among secondary school teachers in Nigeria (30.9%),^8^ Kenya (32.7%),^38^ and Ghana (23.2%).^6^ These differences could be attributed to variations in beliefs, religious and cultural practices, and regulation regarding alcohol use.^4^ In addition, relative to the prevalence reported in Ethiopian-based study, the prevalence was lower than that of the general population in rural Sodo district (21.3%).^27^ Although the tools used to screen for HAC were the same (FAST), differences in the study population and teachers’ level of education may be implicated.

The prevalence found in this study was higher than those reported among the general population in Addis Ababa in Ethiopia (2.7%)^39^ and South Africa (9%).^40^ This could be due to differences in the study population and screening tools used.

Male teachers had 4.46 times greater odds of HAC than women. This study supports the findings of male teachers consuming more alcohol in other countries, such as Nigeria,^8^ Ghana,^6^ and Uganda.^5^ Women in Sub-Saharan Africa perceived more social and cultural sanctions for drinking alcohol, and they are not expected to involve in HAC. Risky behaviors, such as excessive alcohol consumption, are common among men and increase aggression and the risk of physically assault. Alcohol is a key risk factor for sexual violence perpetration. Men are more than 3 times as likely to die by suicide than women and more likely to have been drinking prior to suicide.^41^

Younger teachers, aged <41 years, had 3.76 times greater odds of HAC than older teachers. This finding is consistent with prior findings in Nigeria^8^ and Ghana.^6^ This could be due to drinking habits established in adolescence continuing into young adulthood but then decreasing with age, as responsibilities increase. In addition, aging can lower the body’s tolerance for alcohol. This means that older adults generally experience the effects of alcohol more quickly than when they were younger.^27^^,^^36^

In this study, the authors found a significant association between lower education levels and HAC. Teachers with only a bachelor’s degree had 2.72 times higher odds of HAC than those with advanced degree. This means that higher education status was protective against HAC. There are contrasting findings related to the level of education and HAC. Similar prior findings justified that higher level of education increases the chance to access information on the adverse effects of HAC,^8^^,^^42^ and educated people will prefer healthy lifestyle habits, avoiding risky behaviors. However, other prior studies that are against this finding found that excessive drinking behavior increases with increment in level of education.^43^

Participants who screened positive for psychological distress had 4.46 times higher odds of HAC than those who screened negative. This finding is consistent with studies reported on distress elsewhere. People who have underlying psychological problems are commonly more prone to drinking large quantities of alcohol, possibly as self-medication agents, or HAC may predispose users to psychological distress.^44^^,^^45^

Teachers with low social support had 3.95 times higher odds of HAC compared with those with strong social support, even though many were married and living with family. This underscore that the mere presence of familial ties does not ensure protective support, and quality of social relationships matters more than their existence.^46^ Teachers are at risk for poor social support due to factors such as professional isolation, emotional burden, and cultural norms, turning to alcohol as coping mechanism, thereby raising the risk of HAC.^46^

### Limitations

Limitations of this study include recall bias. In addition, the results may be influenced by response bias. Because this study used a cross-sectional design, the findings do not indicate whether the relationship between psychological distress and HAC is causal.

## CONCLUSIONS

High levels of HAC were observed in teachers in Ethiopia, particularly compared with previous literature of HAC in the general population in Ethiopia. Furthermore, HAC in teachers was associated with several characteristics, specifically being male, being younger in age (<41 years), having lower education levels, having limited social supported, and experiencing psychological distress. The finding refers for significant attention of stakeholders to strengthening lifestyle and other psychosocial interventions in the teacher’s development programs.
